# Real-World Adherence to a Delirium Screening Test Administered by Nurses and Medical Staff during Routine Patient Care

**DOI:** 10.3390/brainsci14090862

**Published:** 2024-08-27

**Authors:** Rashad Soboh, Meital Rotfeld, Sharon Gino-Moor, Nizar Jiries, Shira Ginsberg, Ron Oliven

**Affiliations:** 1 Fliman Geriatric Hospital, Haifa 31021, Israel; 2 Geriatric Unit, Bnai Zion Medical Center, Haifa 3339419, Israel; 3 Department of Medicine, Bnai-Zion Medical Center, Haifa 3339419, Israel; 4 Rappaport School of Medicine, Technion Institute of Technology, Haifa 3109601, Israel

**Keywords:** delirium, screening test, adherence, cognition, RADAR

## Abstract

Delirium is often the first symptom of incipient acute illness or complications and must therefore be detected promptly. Nevertheless, routine screening for delirium in acute care hospital wards is often inadequate. We recently implemented a simple, user-friendly delirium screening test (RMA) that can be administered during ward rounds and routine nursing care. The test was found to be non-inferior to 4AT in terms of sensitivity and specificity. However, the dominant factors to take into account when assessing the performance of a test added to the routine work of busy acute care hospital wards are ease of administration, real-life amenability and the ability of the staff to adhere to testing requirements. In this study, we evaluated the prevalence of daily RMA tests that were not administered as scheduled and the impact of these omissions on the overall real-world performance of RMA. Using point-in-time assessments of 4AT by an external rater, we found that complete RMA was administered in 88.8% of the days. Physicians omitted significantly more tests than nurses, but their results were more specific for delirium. Omissions reduced the sensitivity and specificity of RMA for delirium (compared to 4AT) from 90.7% to 81.7%, and from 99.2% to 87.8%, respectively. Ideally, the number of omitted RMA tests should be minimized. However, if over 85% of the daily quota of complete tests are administered, the sensitivity and specificity of RMA for diagnosing delirium as soon as it appears remain at acceptable levels.

## 1. Introduction

Delirium is an acute deterioration in mental functions characterized by a disturbance in cognitive function, awareness, attention, and with an acute onset and fluctuating course. It results from the interaction of precipitating causes such as drugs, infection, and surgery combined with predisposing factors such as age and dementia [1,2]. Delirium is common in hospitalized geriatric patients, and affects more than a quarter of all patients in internal medicine wards aged 70 years and over [3,4,5,6]. Delirium increases mortality [3] increases the length of hospital stays [5], and increases the likelihood of persistent cognitive deficits [5] and discharge to a higher level of care [1,6].

Given the heavy financial and healthcare burden of delirium, early detection remains critical in any institutional care setting [7]. Routine daily screening for delirium may be particularly important in the non-surgical setting because mental deterioration in older patients can be the first sign of disorders and diseases that precipitate delirium [8,9,10,11]. Nevertheless, despite the development of several “ultra-brief” tests [12,13,14,15,16,17,18,19], delirium remains under-diagnosed [20,21] for several reasons: lack of awareness of its importance, confusion between delirium and dementia, and the absence of in-service continuous education and staff training [22,23].

We believe that in order to improve the recognition and management of delirium, the ward’s staff should be actively involved in the process of identifying this disorder. This approach is most useful for improving the knowledge, awareness and understanding of the significance of delirium. Dividing screening duties between nurses and physicians is beneficial both for sharing the awareness and responsibility and for reducing the workload of each. The test can be administered in a few minutes, which improves compliance; it eliminates the need for a dedicated “delirium diagnostician”; and above all, it allows both nurses and physicians to immediately implement the measures needed in patients with delirium.

For this purpose, we combined two well-known daily cognitive impairment (CI) tests: Recognizing Acute Delirium As part of your Routine (RADAR) [24,25,26], which is administered by nurses to all patients aged 70 years and older; and the Months Of the Year Backwards (MOYB) test [27], which is administered by physicians to patients with an abnormal RADAR. Delirium is diagnosed if both RADAR and MOYB are abnormal and the CI is confirmed to be new. We call this test, which combines RADAR + MOYB + Acute, RMA.

In a previous study [28], we compared the results obtained from complete RMA tests correctly administered by ward staff, with those obtained from the 4AT administered by an external rater, and found a high concordance [28]. The 4AT, a widely used test for delirium, is based on four items: Alertness, AMT4 (Abbreviated Mental Test), Attention (MOYB) and Acute change or fluctuating course. It was chosen because it is very similar to the RMA test and was validated most thoroughly. Therefore, a high concordance between 4AT and RMA obviated the need to perform more complicated tests to confirm the validity of RMA.

In the real world, however, the sensitivity and specificity of a test administered by the busy staff of an acute internal medicine ward relies far more on compliance and adherence to guidelines and the ability of the staff to administer the test during their daily rounds. Failing to administer tests as required, either by nurses or physicians, can have a far greater impact on the quality and validity of the test than false positive or negative results [28,29]. Nevertheless, almost no studies have evaluated adherence to daily delirium screening by the departmental stuff during routine work. In a recent study from New Zealand, 4AT was integrated into doctors’ electronic admission forms as a mandatory assessment. Adherence to this test, performed only on admission, reached 83.2% [30]. In a more recent study from Brazil, the percentage of admissions screened by nurses for delirium using the CAM test reached 98.7%, but adherence to daily screening was achieved only in 24.5% of admissions [31].

The purpose of the present study was to assess the “true”, real-life RMA test efficacy for detecting delirium, also taking into account the days when the RMA tests were not administered as required. For this purpose, we administered 4AT arbitrarily a few days a week in addition to the daily RMA tests, starting a year after RMA was included and adequately integrated into the department’s routine. This approach enabled us to assess the feasibility and efficacy of the basic concept of daily screening for delirium by nurses and physicians during their routine work while incorporating the main problem of this idea—incomplete adherence of the staff for whom delirium detection is not the only or main task. In addition, we compared the sensitivity and specificity of RADAR to those of RMA to assess the contribution of the physicians to the diagnosis of delirium, and to compare their adherence to the RMA requirements to that of the nurses.

## 2. Methods

We report the results of a retrospective analysis of all consecutive patient records that included the 4AT over 6 months. This study was approved by the Human Institutional Review Board of Bnai Zion Medical Center, Haifa, Israel.

Subjects: Patients aged 70 years and over, hospitalized in internal medicine wards in the Bnai-Zion Medical Center, a municipal hospital affiliated with the faculty of medicine were included in this study. Patients with a diagnosis of severe dementia referred from nursing homes, terminally ill patients, and patients unable to cooperate due to language barriers were excluded.

Delirium screening: A. RMA. Before starting the present study, all nurses and residents were given comprehensive instructions and training on the administration of the test. RMA fields were added to electronic medical records used by both nurses and doctors (see below). For the first 6 months following implementation, geriatric nurses performed spot checks on the RMA records to verify they were filled out as required. Following this, staff from the central nursing office performed spot checks on the nurses’ records and the geriatric unit staff performed spot checks on the physicians’ records.

The RMA test was administered daily to each patient by the ward staff during the morning rounds, in 3 stages: first, RADAR was administered to all older patients (age > 69 years) by nurses based on their observation of the patient while dispensing medication, as previously described [22,23]. In the electronic medical records, nurses answered “yes” or “no” to 3 questions: 1. Was the patient unusually sleepy? 2. Did the patient have difficulty following instructions? 3. Were the patient’s movements unusually slow? If the answer was “yes” to one or more of the questions, the RADAR test was considered positive and a red flag appeared in the resident’s field of the patient’s electronic medical records, indicating possible CI.

In the next step, all patients with records indicating a positive RADAR test were evaluated by the treating physician using MOYB during their routine morning rounds. MOYB was administered within 3 h of RADAR, and performed as in 4AT (see below), but scored only as positive or negative. A negative MOYB confirmed the presence of an attention/concentration deficit, and was used, together with the nurse’s assessment (i.e., positive RADAR), to confirm the presence of a CI.

Finally, the CI was defined as “chronic stable” (chronic cognitive impairment [CCI]), usually dementia) or “new onset”, including fluctuating cognition, indicating delirium. Delirium superimposed on dementia was scored as delirium. The recency of CI was based on information provided by the patient’s relatives, friends or caregivers on the first day of hospitalization. After that, it was based on the follow-up and tests administered over the previous days. The RMA was only positive for delirium if both RADAR and MOYB were positive and the CI was new.

B. 4AT. For the purpose of this study, the administration of the 4AT started 1 year after RMA had been included in the department’s routine work. A detailed description of the parameters and scoring of 4AT is available in https://www.the4at.com (accessed on 25 August 2024). 4AT was used to verify the reliability of RMA and was administered by a resident in geriatrics with extensive experience with this test. It was administered as previously described [12,13].

Unlike RMA, the 4AT was administered arbitrarily 2–4 times a week (i.e., not every day and not in every patient) on weekdays, at noon, within 4 h of RADAR. In a given patient, the 4AT was administered once a day, either once or a few times, on consecutive or non-consecutive days. In order to prevent bias, the 4AT scorer had no access to the ward staff’s RMA results; so, he was unaware of whether both RADAR and MOYB had been administered before the 4AT. Furthermore, the 4AT scorer had no access to the patient’s records; so, if CI was diagnosed on the basis of the first 3 As, he was unable to determine whether the patient’s CI was new or pre-existing. Since this information could only be obtained by the ward physician, the existence of new vs. pre-existing CI was later retrieved from the RMA test, and was always equal in both tests.

The results of the 4AT, the “gold-standard” in this study, were also entered into the patient’s medical records. A 4AT score of 0–1 was considered normal, and a score ≥ 2 indicated the presence of CI. A new or fluctuating course, one of the criteria of the 4AT, that provides a score of 4, was mandatory for the identification of delirium. Therefore, a score of at least 6 was required for the diagnosis of delirium by 4AT.

Data analysis. Data from the days in which the 4AT was administered were extracted from the medical records retrospectively after 6 months. The results are presented in binary form—positive or negative for delirium—but the actual 4AT score was also available. In addition to new onset or fluctuating course, both positive RADAR and MOYB were required for the RMA test to be positive for delirium. The sample size required for a sensitivity of 90% and a 95% confidence interval, calculated according to Buderer et al. [32], was more than 304 subjects, assuming a delirium prevalence of >20% in older patients in internal medicine wards [3]. RMA sensitivity, specificity, positive and negative predictive values, and inter-test concordance [(true positive +true negative)/n], the phi coefficient (measure of the degree of association between 2 binary variables) and the probability of agreement (Cohen’s kappa) were used to compare RMA and 4AT.

## 3. Results

The 4AT was administered on 562 days, and the data presented refer to information obtained on those days. The 4ATs were administered more than once to 128 patients on several [2,3,4,5,6] days, and only once to the other 239 patients. Since 4ATs administered repeatedly to the same patient were often administered on non-consecutive days and sometimes also in different admissions, the following data analysis refers to the 562 days in which 4AT was administered.

**Figure 1:**
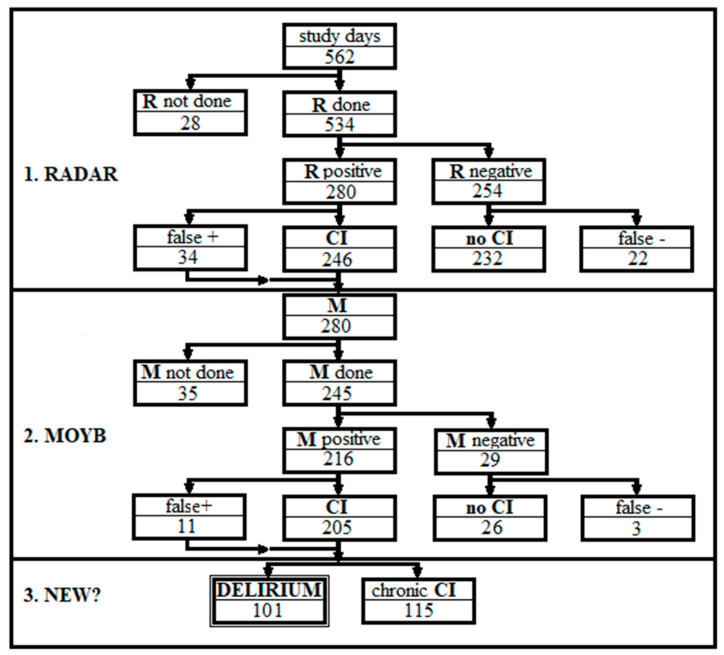
Flow-chart of this study. The numbers indicate the sum of tests in each stage according to the RMA protocol. R—RADAR; M—MOYB; CI—cognitive impairment; NEW?—is the CI new, i.e., delirium?

Figure 1 shows this study’s flow-chart. The numbers show the sum of RMA tests in each stage according to the RMA protocol. Although the RMA test is quite simple, in order to analyze the data, it was necessary to divide the patients into groups (with and without CCI or delirium), to address staff errors (false positive and negative based on 4AT findings), and to take into account tests that were omitted by nurses or physicians. The nurses failed to administer RADAR on 28 days (5.0%), while physicians did not administer MOYB on 34 required days (12.1%, *p* < 0.03 compared to nurses’ omissions). Accordingly, complete RMA tests were administered on 499 of the 562 study days (88.8%). Nurses had false positive and false negative results on 34 and 22 days, respectively. In other words, nurses wrongly assessed 8.4% of patients considered by 4AT to have CI. In comparison, physicians found false positive and false negative MOYB on 4 and 3 days, respectively (misdiagnosis rate of 2.8%, *p* < 0.01 compared to nurses). On three occasions, they incorrectly overruled the nurse’s correct assessment of CI, but also overruled 30 false positive RADARs. Based on the RMA protocol, false positive RMA tests were possible only when both nurse and residents considered the patient to be delirious but the 4AT scorer found no signs of CI.

Table 1 shows the age and gender of the patients and the 4AT results on all days. The male–female distribution (62.1% women) was similar in patients with delirium, CCI and without CI. Patients with delirium were significantly older than those without CI (84.9 ± 6.8 vs. 80.5 ± 6.6, *p* < 0.001). On 294 days (52.3%), the 4AT score was 0 or 1, defined in this study as no CI. A CI identified as new (i.e., delirium) was verified by 4AT on 120 days (21.4%). On the remaining days, the CI was considered to be pre-existing (CCI, probably dementia). Due to the four-point score given to new-onset CI, the average 4AT score on days with delirium was significantly higher than that of days scored in patients with CCI. However, even after deducting the four points, the average score on delirium days remained significantly higher (*p* < 0.03).

Using the RMA test, the staff diagnosed delirium on 101 days, with 97 true and 4 false positive results on the days when both RADAR and MOYB were administered within the specified time frame. The results of the RMA correlated highly with those of the 4AT, with a phi coefficient of association of 0.91 (*p* < 0.001). Concordance between the 4AT and RMA assessments was 97.4%, and the calculated free marginal kappa was 0.91 (confidence interval 0.88–0.95).

In a test such as the RMA that is administered by staff daily during their rounds, compliance and adherence to the protocol is the most important factor. The staff’s efficacy in detecting delirium largely depends on the number of days in which the test was omitted for whatever reason. Table 2 shows the sensitivity, specificity, and negative and positive predictive values of the RMA and the level of concordance between the 4AT and RMA (and separately between 4AT and RADAR) without (i.e., only days in which RMA and RADAR were performed, upper rows of RMA and RADAR in the table) and after including the days in which testing was omitted by the staff. All omitted days were considered false negatives, and were added as such to the RMA and RADAR analysis (lower rows in the table). RMA sensitivity was over 90%, and all other parameters were well over 95% for the days in which RMA was administered. However, all parameters decreased considerably after including the days in which RMA was omitted by nurses or physicians (with the exception of PPV, which is based, by definition, on administered RMA data). The sensitivity of RADAR was slightly higher than that of RMA due to the few additional false negative findings introduced by the physicians. However, as RADAR assesses only CI, its specificity for delirium is low. Taking into account the omitted days further worsened all RADAR parameters.

In the 128 patients in whom the 4AT was administered more than once (2.5 ± 1.0 mean ± SD days/patient, total of 323 days), delirium was found in 30 patients, and in 16 of these cases, a transition between delirium and a non-delirium state was diagnosed by 4AT. RMA recognized correctly 12 of these transitions. It was incomplete on 2 days, but delirium was diagnosed on a subsequent day. In another two patients, the development of delirium was overlooked (false negative RMA).

In order to determine the characteristics of the 63 patients for whom the required RMA tests were omitted, we compared these patients to the rest of the cohort (Table 3). No significant difference was found between the groups, except for a significantly higher prevalence of benzodiazepine treatment in patients in whom RMA was not administered. Of note, due to the far higher number of patients in whom RMA was administered, the actual number of patients treated with these drugs was more than three times higher in the general cohort (10/63 = 15.9% and 32/499 = 6.4%, respectively).

## 4. Discussion

In this article, we describe our experience using the RMA test administered jointly by nurses and physicians during their routine work to detect delirium in geriatric patients hospitalized in acute care internal medicine wards. Our main findings are as follows: 1. After adequate planning and preparations, and teaching and practicing the subject, acceptable delirium screening on a daily basis by the department’s stuff during their routine work is feasible. 2. The level of adherence in performing RMA tests significantly affects the reliability of the test, but after implementing the RMA test into the daily work routine, and maintaining a low-grade supervision, the percentage of missed tests can be reduced to a reasonable level. 3. The RADAR test provides a high sensitivity to the screening for CI, but adding the evaluation of the physician and confirmation of the recentness of CI significantly improves the specificity of RMA for delirium.

Although the significance of prompt recognition of emerging delirium is well established, delirium diagnosis in acute care hospital wards is often unsatisfactory. Validated delirium recognition tests with high sensitivity and specificity, such as CAM and 4AT, have been developed, but few hospital administrators are willing to employ dedicated healthcare personnel to administer daily delirium tests. Even appropriately trained physicians are often reluctant to add the few minutes required to administer these tests to every geriatric patient to their busy schedule. To reduce the number of patients requiring delirium assessment by clinicians, short preliminary screening tests [16,17,18,24,25,26] that can be administered by nurses were developed. In this approach, patients testing positive in the preliminary screening would then undergo a complete test by a professional. It is unclear to what extent this approach has gained acceptance in hospitals. We believe that the RMA test, a simple, easily administered yet accurate delirium screening tool that can be administered by acute care ward staff during routine work, is likely to gain acceptance and reasonable adherence in many hospitals.

As expected, based on the findings of our previous study [28], the fully completed RMA (i.e., no missing tests) showed a high correlation with the 4AT. We chose the extensively validated 4AT [12,33] as a comparator because of its similarity with RMA: assessment of alertness (the first “A” of 4AT) is performed in RMA by the RADAR. Although this item was evaluated by a rater-physician in 4AT and by nurses in RMA, a previous comparison showed that physicians and nurses tend to rate delirium similarly [19]; also, MOYB is used in both tests, and the fourth “A”, the presence of an acute or fluctuating course of CI, was the same in both tests. The main difference between tests is that the AMT4 is not included in the RMA test. In contrast to 4AT, the RMA was administered every day by staff who were already familiar with most patients, making it easier for them to perceive a change in the patent’s cognitive state. Although we used 4AT as the gold standard, it has been found to have a pooled sensitivity and specificity of 0.88 [33]. Therefore, one may assume that the assessments of the staff were sometimes more accurate than those of the rater, who usually met the patients for the first time when administering the 4AT.

The real-world efficacy of the RMA test, i.e., taking into account tests omitted by the staff, was assessed by comparing its results to those obtained by an external assessor who performed random spot checks using the 4AT. False positive results are of minor importance: false positive RADAR can be corrected by physicians, and only 3 out of the 11 patients with false positive MOYB had new CI, i.e., delirium. However, omissions had a major effect on sensitivity, specificity, and NPV. The number of patients with undiagnosed delirium due to omitted tests exceeded those with false negative results (12 and 10 patients, respectively). Therefore, in a delirium screening test administered by staff during their routine work (rather than by a dedicated assessor), minimizing omissions by improving compliance and adherence to rules may be more important in practice than improving diagnostic accuracy. It should be noted that some of the inconsistencies between RMA and 4AT could have resulted from fluctuating changes in the state of consciousness within the few hours between tests, i.e., the true false negative results may have been lower than found in this study.

Given both the ubiquity and negative sequelae of delirium and the growing evidence of the effectiveness of delirium prevention and management, screening is an essential part of hospital care and a prerequisite for proper management [34]. It should be emphasized that implementing routine screening of delirium is not a simple task. In our hospital, both the head nurse’s office and the geriatric unit recognized the significance of this issue, and were committed to implementing adequate screening and management of delirium. Several measures were introduced to achieve this goal: the RMA test was developed and validated by comparing it to 4AT; both nurses and physicians were thoroughly trained in the use of the tests, and received occasional refresher courses and reminders at staff meetings; dedicated RMA fields (RADAR for nurses, MOYB and recentness of CI for physicians), as well as a 4AT field were added to the electronic medical record; point-in-time 4AT screening by an external assessor was added for a limited period; and spot checks were performed by the nursing office and the geriatric unit. In these spot checks, we found that the staff’s adherence to the RMA test improved fast initially, and gradually reached a steady state 9–10 months after implementation. Therefore, we chose to start collecting the data required to evaluate the adherence achieved only a year after the implementation of RMA. At that time, we had overcome some of the major barriers to diagnosing delirium—insufficient awareness, lack of adequate education, and lack of knowledge of how to diagnose delirium [23,35]. This conclusion is based on the high specificity of the RMA when it was completed as required. Although the test’s sensitivity (based mainly on the nurse’s impression) could be improved, it should be noted that the RADAR false negative rate was lower than 4%. Accordingly, to improve the sensitivity and specificity of RMA, efforts should be focused on improving adherence and reducing the number of missed tests.

Poor adherence to instructions was usually attributed to the staff’s heavy workload. However, in the case of a brief test that is only needed in some of the patients (RADAR only in the older patients, MOYB only in those with a positive RADAR), overwork can only justify a few omitted tests. It was suggested to view RADAR as an additional vital sign, and, in fact, nurse adherence to testing was found to be high. An unexpected finding was that many of the patients that were not assessed with RMA were treated with benzodiazepines (Table 3). Apparently, RMA was occasionally omitted in patients who appeared drowsy in the morning and were known to be under the influence of sleep medication.

This study also allowed us to assess the independent efficacy of the RADAR test since one may argue that the assessment of the nurses may be sufficient, making the physicians’ assessment redundant. As shown in Table 2, although the sensitivity of RADAR was slightly higher than that of RMA, we found that the physician component (MOYB and assessment of CI recency) is important to improve the specificity of RMA for delirium, as positive RADAR is a sensitive tool to detect the presence of CI but does not indicate delirium. For delirium, confirmation of the recency of CI is needed. This has to be confirmed by other means, not during medication rounds. Including physicians in the diagnostic procedure has additional goals: it increases their awareness of this often-overlooked complication; improves their adherence to diagnostic and therapeutic guidelines for patients with delirium; and in addition to improving the specificity of RMA, corroboration of the presence of inadequate attention/concentration (provided by MOYB) is included in the DSM-5 definition of delirium [36].

Several limitations of this study need to be addressed. First, it can be assumed that the causes and prevalence of omitted delirium diagnostic tests are largely related to the specific circumstances in certain departments and/or hospitals. The adherence data presented here only reflect the situation in the internal medicine department of our hospital and cannot be extrapolated to other settings. Therefore, when implementing a delirium screening test to be administered by staff, local issues such as the frequency of administration and reasons for inadequate adherence need to be addressed in order to improve staff cooperation and adherence [34,37,38]. Another shortcoming is that the physician is not required to administer the MOYB test when the nurse reports a negative RADAR. This hinders the physicians’ ability to improve the sensitivity of RMA by detecting cases of delirium that were not recognized by nurses. However, changing this protocol would have required MOYB to be administered in all patients. We choose to accept this shortcoming in order to keep the RMA test user-friendly, and our results show that despite this, the RMA is reasonably accurate. Also, although RMA and 4AT were administered in relatively quick succession, it is possible that short fluctuations in the state of consciousness could have contributed to differences between the test results. In addition, the validity of RMA has so far been assessed only in the department of internal medicine, and its reliability in surgical departments still needs to be confirmed. Finally, the type of delirium (hyper/hypoactive) could affect the adherence, and recognition of delirium could not be evaluated in this study because agitated patients were usually treated before MOYB and 4AT were administered.

## 5. Conclusions

The utilization of RMA test for delirium identification, carried out in collaboration between nurses and physicians during their routine work, has several valuable advantages: it enables routine and regular assessment; it improves the staff’s awareness of delirium and helps train them to accurately diagnose this condition; it avoids placing an additional burden on a single section of staff; and it overcomes communication gaps and delays, as delirium is diagnosed by the same healthcare providers in charge of managing and treating this condition. No screening tool is perfect, and both CAM and 4AT yield false positive and negative results. The success of screening during routine work depends on staff cooperation and adherence to test requirements. Our experience has shown that in the real world, adherence may have a greater impact on the validity of the test than the diagnostic accuracy of the stuff. However, provided omissions did not exceed 10–15%, the real-world sensitivity and specificity of RMA exceed 80% even when taking into account omitted tests.

## Figures and Tables

**Table 1:** Patient characteristics: gender, age and cognitive state as defined by the 4AT. Gender and age refer to the 367 patients. As many of the patients were tested more than once, cognition data are provided for the total number of days in which 4AT and RMA were performed (n = 562).

Patients (n = 367)	Female (%)	62.1
	Age (Years, mean ± SD)	82.3 ± 7.2
study days (n = 562)	Delirium (%) and 4AT score	120 (21.4%), 7.6 ± 1.6
	CCI (%) and 4AT score	148 (26.3%), 2.9 ± 1.9
	no-CI (%), 4AT = 0 or 1	294 (52.3%)

CI—cognitive impairment (CCI + delirium). CCI—chronic unchanged CI (4AT score > 1). Delirium—acute CI, including delirium superimposed on CCI (4AT score > 5). no CI—4AT score 0 or 1.

**Table 2:** Sensitivity, specificity, positive and negative predictive values of RMA and RADAR for delirium, as identified by 4AT. For RMA, parameters were calculated separately for the days in which both RADAR and MOYB (when required) were administered (n = 499) and for all days (n = 562, including the 63 days in which tests were omitted). Similarly, for RADAR, the parameters are those calculated only for the days when RADAR was administered (n = 534), and for all days, including the 28 days when RADAR was omitted.

		Sensitivity (%)	Specificity (%)	PPV (%)	NPV (%)	Agreement (%)
RMA	test administered n = 499	90.7	99.2	97.0	97.5	97.4
	all days n = 562	81.7	87.8	97.0	84.2	88.2
RADAR	test administered n = 534	93.1	58.6	38.6	96.8	66.3
	all days n = 562	90.0	61.1	38.6	87.9	67.3

PPV, NPV—positive and negative predictive value; agreement—concordance between 4AT and RMA or RADAR ([true positive + true negative]/n).

**Table 3:** Parameters of patients in whom a complete RMA assessment was not performed.

	RMA Not Performed n = 63	RMA Performed n = 499
Age (years)	83.8	82.1
Gender (% female)	65.4	61.6
Delirium (%)	19.0	21.6
no delirium (%)	81.0	78.4
Benzodiazepines (%)	15.9	6.4 *
Anti-psychotics (%)	3.4	3.5
Fever (>37.5) (%)	6.8	3.6
Urinary retention (%)	0	4.0

CI—cognitive impairment (4AT > 1). * *p* < 0.005

## Data Availability

The data presented in this study are available on request from the corresponding author due to ethical reasons.

## References

[1] Vidal E.I., Villas Boas P.J., Valle A.P., Cerqueira A.T., Fukushima F.B. (2013). Delirium in Older Adults. BMJ.

[2] Marcantonio E.R. (2017). Delirium in Hospitalized Older Adults. N. Engl. J. Med..

[3] Hshieh T.T., Inouye S.K., Oh E.S. (2018). Delirium in the Elderly. Psychiatr. Clin. N. Am..

[4] Jackson T.A., MacLullich A.M., Gladman J.R.F., Lord J.M., Sheehan B. (2016). Undiagnosed Long-term Cognitive Impairment in Acutely Hospitalized Older Medical Patients with Delirium: A Prospective Cohort Study. Age Ageing.

[5] Oh E.S., Fong T.G., Hshieh T., Inouye S.K. (2017). Delirium in Older Persons: Advances in Diagnosis and Treatment. JAMA.

[6] Huang L.W., Inouye S.K., Jones R.N., Fong T.G., Rudolph J.L., O’Connor M.G., Metzger E.D., Crane P.K., Marcantonio E.R. (2012). Identifying Indicators of Important Diagnostic Features of Delirium. J. Am. Geriatr. Soc..

[7] Inouye S.K., Bogardus S.T., Charpentier P.A., Leo-Summers L., Acampora D., Holford T.R., Cooney L.M. (1999). A Multicomponent Intervention to prevent Delirium in Hospitalized Older Patients. N. Engl. J. Med..

[8] Hshieh T.T., Yue J., Oh E.S., Puelle M., Dowal S., Travison T., Inouye S.K. (2015). Effectiveness of Multicomponent Nonpharmacological Delirium Interventions: A Meta-analysis. JAMA Intern. Med..

[9] Hshieh T.T., Yang T., Gartaganis S.L., Yue J., Inouye S.K. (2018). Hospital Elder Life Program: Systematic Review and Meta-Analysis of Effectiveness. Am. J. Geriatr. Psychiatry.

[10] Zazzara M.B., Penfold R.S., Roberts A.L., Lee K.A., Dooley H., Sudre C.H., Welch C., Bowyer R.C.E., Visconti A., Mangino M. (2020). Probable Delirium is a Presenting Symptom of COVID-19 in Frail, Older Adults: A Cohort Study of 322 Hospitalised and 535 Community-based Older Adults. Age Ageing.

[11] Zoremba N., Coburn M. (2019). Acute Confusional States in Hospital. Dtsch. Arztebl. Int..

[12] Bellelli G., Morandi A., Davis D.H.J., Mazzola P., Turco R., Gentile S., Ryan T., Cash H., Guerini F., Torpilliesi T. (2014). Validation of the 4AT, a New Instrument for Rapid Delirium Screening: A Study in 234 Hospitalized Older People. Age Aging.

[13] MacLullich A.M., Shenkin S.D., Goodacre S., Godfrey M., Hanley J., Stíobhairt A., Lavender E., Boyd J., Stephen J., Weir C. (2019). The 4′A’s Test for Detecting Delirium in Acute Medical Patients: A Diagnostic Accuracy Study. Health Technol. Assess..

[14] Marcantonio E.R., Ngo L.H., O’Connor M., Jones R.N., Crane P.K., Metzger E.D., Inouye S.K. (2014). 3D-CAM: Derivation and Validation of a 3 Minute Diagnostic Interview for CAM-defined Delirium. Ann. Intern. Med..

[15] Motyl C.M., Ngo L.H., Zhou W., Jung Y., Leslie D., Boltz M., Husser E., Inouye S.K., Fick D.M., Marcantonio E.R. (2020). Comparative Accuracy and Efficiency of Four Delirium Screening Protocols. J. Am. Geriatr. Soc..

[16] Sillner A.Y., Ngo L.H., Jung Y., Inouye S.K., Boltz M., Leslie D., Marcantonio E.R., Fick D.M. (2020). Ultrabrief Screens for Detecting Delirium in Postoperative Cognitively Intact Older Adults. J. Hosp. Med..

[17] Powers J.S., Doering T., Gordon S., Eden S.K., Shintani A., Schnelle J. (2013). Exploring the Utility of Ultra-brief Delirium Assessments in a Nonintensive-Care Geriatric Population: The GEM Study. Gerontologist.

[18] Fick D.M., Inouye S.K., Guess J., Ngo L.H., Jones R.N., Saczynski J.S., Marcantonio E.R. (2015). Preliminary Development of an Ultrabrief Two-item Bedside Test for Delirium. J. Hosp. Med..

[19] Fick D.M., Inouye S.K., McDermott C., Zhou W., Ngo L.H., Gallagher J., McDowell J., Penrod J., Siuta J., Covaleski T. (2018). Pilot Study of a Two-step Delirium Detection Protocol Administered by Certified Nursing Assistants, Physicians and Registered Nurses. J. Gerontol. Nurs..

[20] Inouye S.K., Rushing J.T., Foreman M.D., Palmer R.M., Pompei P. (1998). Does Delirium Contribute to Poor Hospital Outcomes? A three-site Epidemiologic Study. J. Gen. Intern. Med..

[21] Cole M.G., Fenton F.R., Englesmann F., Mansouri I. (1991). Effectiveness of Geriatric Psychiatry Consultation in an Acute Care Hospital: A Randomized Clinical Trial. J. Am. Geriatr. Soc..

[22] Hogan D.B., Fox R.A., Gadley B.W.D., Mann O.E. (1984). Effect of a Geriatric Consultation Service on Management of Patients in an Acute Care Hospital. CMAJ.

[23] Teodorczuk A., Reynish E., Milisen K. (2012). Improving Recognition of Delirium in Clinical Practice: A Call for Action. BMC Geriatr..

[24] Voyer P., Champoux N., Desrosiers J., Landreville P., McCusker J., Monette J., Savoie M., Richard S., Carmichael P.H. (2015). Recognizing Acute Delirium as Part of your Routine [RADAR]: A Validation Study. BMC Nurs..

[25] Voyer P., Champoux N., Desrosiers J., Landreville P., McCusker J., Monette J., Savoie M., Carmichael P.H., Richard H., Richard S. (2016). RADAR: A Measure of the Sixth Vital Sign?. Clin. Nurs. Res..

[26] Bilodeau C., Voyer P. (2017). RADAR: New Empirical Evidence that Supports its Validity and Relevance for Delirium Screening. NPG.

[27] Meagher J., Leonard M., Donoghue L., O’Regan N., Timmons S., Exton C., Cullen W., Dunne C., Adamis D., Maclullich A.J. (2015). Months Backward Test: A Review of Its Use in Clinical Studies. World J. Psychiatr..

[28] Soboh R., Gino-Moor S., Jiries N., Ginsberg S., Oliven R. (2024). Validation of a Viable Delirium Detection Test Performed by Nurses and Physicians during Routine Patient Care. BMC Geriatr..

[29] Oliven R., Rotfeld M., Gino-Moor S., Schiff E., Odeh M., Gil E. (2021). Early Detection and Intervention for Patients with Delirium Admitted to the Department of Internal Medicine: Lessons from a Pilot Initiative. Dement. Geriatr. Cogn. Disord. Extra..

[30] Alhaidari A.A.O., Matsis K.P. (2022). Barriers to Completing the 4AT for Delirium and its Clinical Implementation in two Hospitals: A Mixed-methods Study. Eur. Ger. Med..

[31] Avelino-Silva T.J., Bittencourt J.A.S., Miguel C.M., da Costa Rozzino T.P., Vaccari A.M.H., Barbosa M.S., Szlejf C. (2023). The Confusion Assessment Method in Action: Implementation of a Protocol to Increase Delirium Screening and Diagnosis. Geriatr. Nurs..

[32] Buderer N.M. (1996). Statistical Methodology: Incorporating the Prevalence of Disease into the Sample Size Calculation for Sensitivity and Specificity. Acad Emerg. Med..

[33] Tieges J., Maclullich A.M.J., Anand A., Brookes C., Cassarino M., O’connor M., Ryan D., Saller T., Arora R.C., Chang Y. (2021). Diagnostic Accuracy of the 4AT for Delirium Detection in Older Adults: Systematic Review and Meta-analysis. Age Ageing.

[34] Young J., Murthy L., Westby M., Akunne A., O’Mahony R. (2010). Guideline Development Group: Diagnosis, Prevention, and Management of Delirium: Summary of NICE Guidance. BMJ.

[35] Davis D., MacLullich A.M.J. (2009). Understanding Barriers to Delirium Care: A Multicentre Survey of Knowledge and Attitudes amongst UK Junior Doctors. Age Ageing.

[36] American Psychiatric Association (2013). Diagnostic and Statistical Manual of Mental Disorders.

[37] Yue J., Tabloski P., Dowal S.L., Puelle M.R., Nandan R., Inouye S.K. (2014). NICE to HELP: Operationalizing National Institute for Health and Clinical Excellence Guidelines to Improve Clinical Practice. J. Am. Geriatr. Soc..

[38] American Geriatrics Society Expert Panel on Postoperative Delirium in Older Adults (2015). American Geriatrics Society abstracted clinical practice guideline for postoperative delirium in older adults. J. Am. Geriatr. Soc..

